# Antioxidant *Artemisia princeps* Extract Enhances the Expression of Filaggrin and Loricrin via the AHR/OVOL1 Pathway

**DOI:** 10.3390/ijms18091948

**Published:** 2017-09-11

**Authors:** Akiko Hirano, Masashi Goto, Tsukasa Mitsui, Akiko Hashimoto-Hachiya, Gaku Tsuji, Masutaka Furue

**Affiliations:** 1 Beauty Care R&D, Health & Beauty Company, Sunstar Group, Kamihamuro 5-30-1, Takatsuki, Osaka 569-1044, Japan; masashi.goto@jp.sunstar.com (M.G.); tsukasa.mitsui@jp.sunstar.com (T.M.); 2 Department of Dermatology, Kyushu University, Maidashi 3-1-1, Higashi-ku, Fukuoka 812-8582, Japan; ahachi@dermatol.med.kyushu-u.ac.jp (A.H.-H.); gakku@dermatol.med.kyushu-u.ac.jp (G.T.); furue@dermatol.med.kyushu-u.ac.jp (M.F.)

**Keywords:** *Artemisia princeps* extract, filaggrin, loricrin, aryl hydrocarbon receptor, OVO-like 1, nuclear factor-erythroid 2-related factor-2

## Abstract

The Japanese mugwort, *Artemisia princeps* (*yomogi* in Japanese), has anti-inflammatory and antioxidant effects. Skin care products containing *Artemisia princeps* extract (APE) are known to improve dry skin symptoms in atopic dermatitis. Atopic dry skin is associated with a marked reduction of skin barrier proteins, such as filaggrin (FLG) and loricrin (LOR). Recently, aryl hydrocarbon receptor (AHR), and its downstream transcription factor OVO-like 1 (OVOL1), have been shown to regulate the gene expression of FLG and LOR. The focus of this paper is to evaluate the effects of APE on the AHR/OVOL1/FLG or LOR pathway since they have remained unknown to this point. We first demonstrated that non-cytotoxic concentrations of APE significantly upregulated antioxidant enzymes, NAD(P)H dehydrogenase quinone 1 and heme oxygenase 1, in human keratinocytes. Even at these low concentrations, APE induced nuclear translocation of AHR and significantly upregulated *CYP1A1* (a specific target gene for AHR activation), *FLG*, and *LOR* expression. AHR knockdown downregulated *OVOL1* expression. The APE-induced upregulation of *FLG* and *LOR* was canceled in keratinocytes with AHR or OVOL1 knockdown. In conclusion, antioxidant APE is a potent phytoextract that upregulates *FLG* and *LOR* expression in an AHR/OVOL1-dependent manner and this may underpin the barrier-repairing effects of APE in treating atopic dry skin.

## 1. Introduction

The genus *Artemisia* is a mostly perennial plant distributed in the northern hemisphere and is composed of approximately 250 species. They have a wide range of applications including uses in medicines, food, and spices [1,2]. *Artemisia princeps* (Japanese mugwort or *yomogi*) is cultivated in East Asia and has been used in traditional Asian medicine for the treatment of inflammation, diarrhea, carbuncles, bacterial infection, and circulatory disorders [1,2]. Many studies have demonstrated the anti-atherosclerotic, anti-oxidant, and anti-inflammatory effects of *Artemisia princeps* extract [1,2,3]. Another member of this genus is *Artemisia arborescens*, which is used for the treatment of psoriasis and other skin diseases in southern Italy [4]. *Artemisia princeps* water extract (APE) is capable of inhibiting hind paw edema and vascular permeability induced by the intradermal injection of histamine or serotonin in rats [5]. Moreover, APE-containing cosmetic products, such as body cream, skin lotion, and shampoo, improve desquamation, dryness, itching, and erythema in patients with atopic dermatitis [6].

The mammalian epidermis is composed of stratified squamous keratinocytes that protect the body against hazard caused by environmental factors. During epidermal differentiation, keratinocytes move from the basal to the cornified layer of the epidermis [7]. Skin barrier maturation is accomplished by sequential and coordinated expression of various skin barrier proteins, such as filaggrin (FLG) and loricrin (LOR) [7]. Perturbed barrier function is critical for the development of not only atopic dermatitis but also other allergic disorders, namely, atopic march [8,9,10,11]. In accordance with these observations, FLG and LOR expression levels have been reported to be reduced in lesional and non-lesional skin in atopic dermatitis [12,13]. Topical application of coal tar or soybean tar has long been used for the treatment of inflammatory skin diseases [13,14]. As both remedies actively enhance the expression of FLG and LOR via aryl hydrocarbon receptor (AHR) [13,14], the AHR-mediated upregulation of barrier proteins has attracted increasing attention in skin barrier and inflammation research [15,16,17]. A recent study has also clarified that the ligation of AHR activates the downstream transcription factor OVO-like 1 (OVOL1), which then induces FLG and LOR expression [18].

In general, antioxidant phytochemicals activate and induce the cytoplasmic-to-nuclear translocation of nuclear factor-erythroid 2-related factor-2 (NRF2), which is a master transcription factor for gene expression of antioxidant enzymes such as NAD(P)H dehydrogenase quinone 1 (NQO1) and heme oxygenase 1 (HO1) in keratinocytes [19,20]. Some antioxidant phytochemicals such as soybean tar and *Opuntia ficus-indica* extract activate NRF2 via AHR pathway [14,19,21]. Other antioxidant phytochemicals such as cinnamaldehyde activate NRF2 without activating AHR [19,22].

In this study, we showed that antioxidant APE activates NRF2/NQO1·HO1 pathway and upregulates the expression of FLG and LOR in an AHR/OVOL1-dependent manner.

## 2. Results

### 2.1. APE Upregulated Antioxidant NRF2/NQO1 Pathway in Normal Human Epidermal Keratinocytes

We first examined the cytotoxicity of graded concentrations of APE (up to 1%) for normal human epidermal keratinocytes (NHEK)s. APE at concentrations >0.3% decreased the cell viability of NHEKs (Figure S2). As NHEKs were constantly viable with APE ≤ 0.03%, these concentrations were used throughout the experiments.

**Figure 1:**
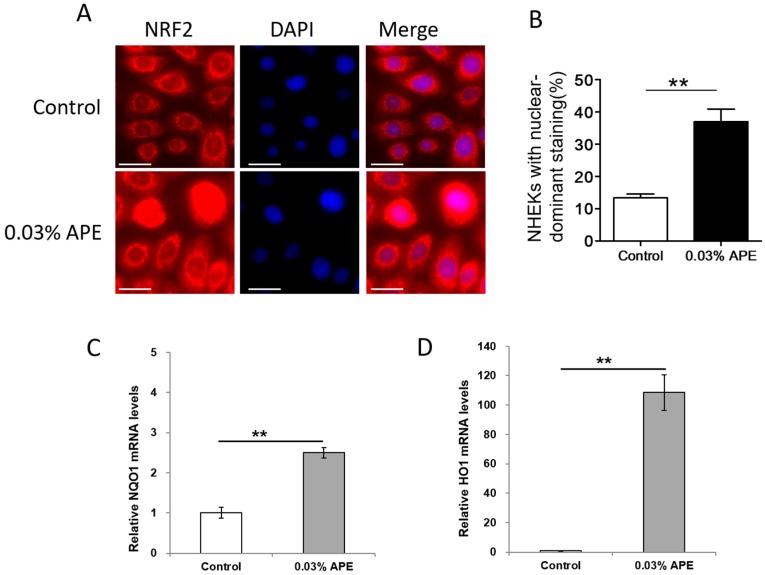
(**A**) Localization of NRF2 is visualized by an immunofluorescence technique. NRF2 is mainly located in the cytoplasm of control keratinocytes. Nuclear staining of NRF2 is enhanced in the keratinocytes treated with APE for 6 h. Nuclei are stained with 4′,6-diamidino-2-phenylindole (DAPI). Scale bar, 20 μm; (**B**) the number of NHEKs with nuclear-predominant staining of NRF2 is significantly increased by APE treatment than that of control; (**C**) APE upregulates the gene expression of antioxidant enzyme *NQO1*; and (**D**) APE also upregulates the gene expression of antioxidant enzyme *HO1*. ** *p* < 0.01.

To prove the biological activity of APE, we next examined whether APE induces the NRF2 activation and the gene expression of antioxidant enzymes, *NQO1* and *HO1*. In control NHEKs, NRF2 was present mainly in the cytoplasm, whereas nuclear staining became enhanced in the presence of APE (Figure 1A). The number of NHEKs with nuclear-predominant staining was significantly increased by APE treatment compared with control (Figure 1B). In parallel with the nuclear translocation of NRF2, APE significantly upregulated the *NQO1* and *HO1* expression (Figure 1C,D), showing that the low concentration of APE did possess an antioxidant property.

### 2.2. APE-Induced AHR Activation in NHEKs

**Figure 2:**
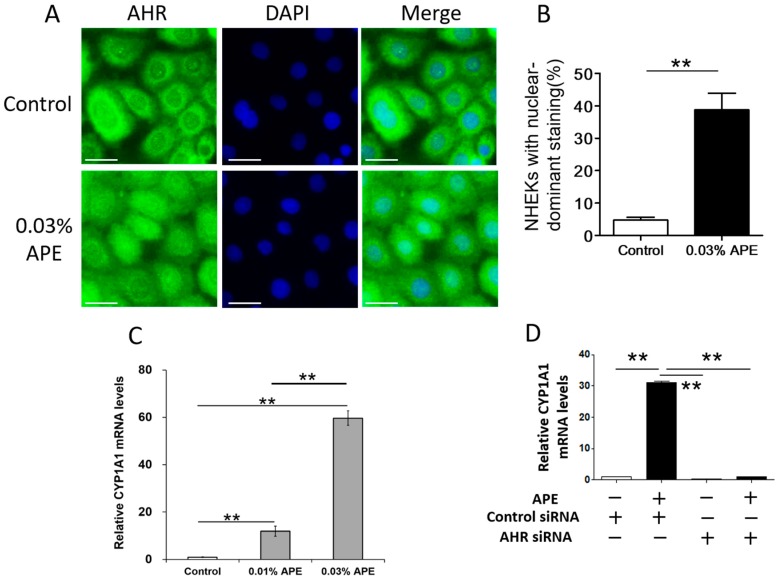
(**A**) Localization of AHR is visualized by an immunofluorescence technique. AHR is mainly located in the cytoplasm of control keratinocytes. Nuclear staining of AHR is enhanced in the keratinocytes treated with APE for 6 h. Nuclei are stained with 4′,6-diamidino-2-phenylindole (DAPI). Scale bar, 20 μm; (**B**) the number of NHEKs with nuclear-predominant staining of AHR is significantly increased by APE treatment than that of control; (**C**) APE dose-dependently upregulates the gene expression of *CYP1A1*, a specific AHR-responsive metabolizing enzyme. ** *p* < 0.01; and (**D**) APE-induced *CYP1A1* upregulation is canceled in keratinocytes transfected with AHR siRNA. ** *p* < 0.01.

As various phytochemicals exert their medicinal effects at least in part by activating the AHR signal [17,21,23], we next examined the AHR-activating capacity of APE. Ligation of AHR is known to induce its cytoplasmic-to-nuclear translocation [13,14,24]. As shown in Figure 2A, AHR was mainly located in the cytoplasm in untreated control NHEKs (upper panel). However, nuclear staining of AHR is enhanced in the keratinocytes treated with APE (lower panel in Figure 2A). The number of NHEKs with nuclear-predominant staining of AHR was significantly increased by APE treatment than that of control (Figure 2B).

AHR activated via nuclear translocation is known to promote the expression of AHR-responsive genes, such as cytochrome P450 1A1 (*CYP1A1*) [24]. As shown in Figure 2C, APE significantly upregulated *CYP1A1* gene expression in a dose-dependent manner. As the gene expression of murine *Cyp1a1* has been shown to be completely abrogated in *Ahr*-null mutant mice [25], we next assessed the AHR dependence of APE-induced *CYP1A1* upregulation using NHEKs transfected with AHR siRNA. The siRNA transfection did not affect cell viability and the inhibitory efficiency of AHR siRNA transfection for AHR mRNA expression was 91.9 ± 2.1%. The APE-induced *CYP1A1* upregulation was canceled in the NHEKs transfected with AHR siRNA (Figure 2D).

### 2.3. APE Induced FLG and LOR Gene Upregulation in an AHR/OVOL1-Dependent Manner

**Figure 3:**
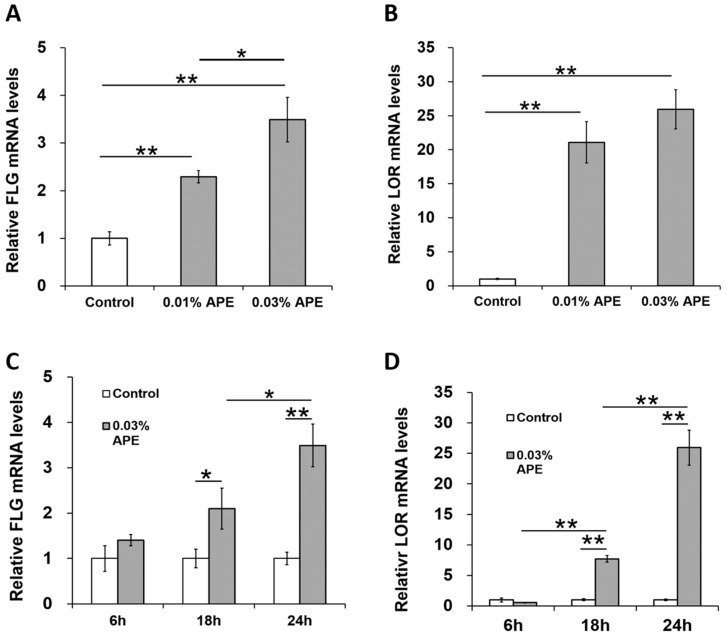
APE enhances the expression of *FLG* (**A**,**C**) and *LOR* (**B**,**D**) in a dose- and time-dependent manner. * *p* < 0.05; ** *p* < 0.01.

A decrease in FLG and LOR expression has been reported to induce barrier disruption and promote skin inflammation [12,13,26]. As AHR signaling upregulates the expression of *FLG* and *LOR* via OVOL1 [13,14,17,18], we next examined whether graded concentrations of APE upregulated *FLG* and *LOR* gene expression. As shown in Figure 3A,B, the levels of *FLG* and *LOR* gene expression were upregulated in the presence of APE. Time course assay revealed time-dependent increases in gene expression for both *FLG* and *LOR* (Figure 3C,D).

In addition, APE also upregulated OVOL1 expression (Figure S3). The APE-induced *OVOL1* upregulation was AHR-dependent because it was canceled in NHEKs transfected with AHR siRNA (Figure S3).

**Figure 4:**
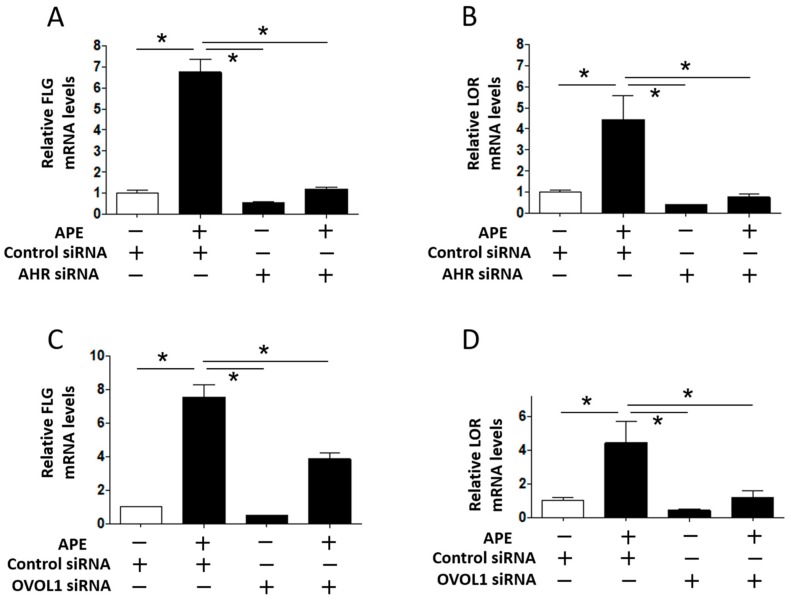
APE (0.03%)-induced *FLG* (**A**) and *LOR* (**B**) upregulation is canceled in keratinocytes with AHR knockdown. APE-induced *FLG* (**C**) and *LOR* (**D**) upregulation is also inhibited in keratinocytes with OVOL1 knockdown. * *p* < 0.05.

To confirm the AHR/OVOL1 dependence of the APE-induced upregulation of *FLG* and *LOR* gene expression, APE was added to NHEKs transfected with AHR siRNA, OVOL1 siRNA, or control siRNA (Figure 4). APE upregulated the *FLG* and *LOR* gene expression in NHEKs transfected with control siRNA, but the enhancing effect was significantly inhibited in NHEKs with AHR knockdown due to transfection with AHR siRNA (Figure 4A,B). Moreover, APE-induced upregulation of *FLG* and *LOR* gene expression was canceled in NHEKs with OVOL1 knockdown compared with that in NHEKs transfected with control siRNA (Figure 4C,D). The inhibitory efficiency of OVOL1 siRNA transfection for OVOL1 mRNA expression was 71.5 ± 2.4% in NHEKs. These results indicate that APE enhanced *FLG* and *LOR* gene expression via the AHR/OVOL1 axis. We finally examined whether APE-induced *NQO1* or *HO1* expression is AHR-dependent or not. As shown in Figure S4, APE-induced *NQO1* expression was only minimally downregulated in NHEKs transfected with AHR siRNA. Moreover, APE-induced *HO1* expression was rather enhanced in AHR-knockdown NHEKs. These results suggest that AHR-independent pathway is mainly operative in APE-mediated NRF2/NQO1·HO1 signaling.

## 3. Discussion

AHR is a xenobiotic chemical sensor that is expressed abundantly in epidermal keratinocytes [15,16]. Various external and internal ligands, such as dioxins, polycyclic aromatic pollutants, benzo[a]pyrene, and phytochemicals, can bind to and activate AHR [15,16]. Upon ligand binding, cytoplasmic AHR undergoes a conformational change and subsequently translocates into the nucleus. The ligand/AHR complex then binds to specific DNA recognition sites, namely, xenobiotic-responsive elements or dioxin-responsive elements, and upregulates the transcription of a series of responsive genes, such as *CYP1A1* leading to a robust generation of reactive oxygen species [16,24]. Various herbal and antioxidant phytochemicals have been shown to activate AHR [16,17,19]. Notably, the AHR-targeting antioxidant phytochemicals or antioxidant medicinal chemicals do not generate appreciable amounts of reactive oxygen species probably due to efficient induction of antioxidant enzymes mediated by NRF2 activation [19,20,21,27].

APE is a widely used herbal extract that has potent anti-inflammatory and antioxidant properties [1,2,3,4,5,6]. In order to understand APE’s molecular mechanisms better, we first revealed the antioxidant property of non-cytotoxic concentrations of APE by demonstrating its action on NRF2/NQO1 and HO1 signaling. The NRF2/NQO1 and HO1 axis is essentially involved in antioxidant activity in keratinocytes [19]. The non-cytotoxic concentration of APE used in the present study did induce the cytoplasmic-to-nuclear translocation of NRF2 and subsequent upregulation of *NQO1* and *HO1* antioxidant enzymes. Even at this low concentration, APE also activated AHR signaling and induced the nuclear translocation of AHR. The ligation of AHR by APE induced the significant upregulation of AHR-specific gene expression of *CYP1A1*.

Skin inflammation decreases FLG and LOR expression and disrupts epidermal barrier function; moreover, in the opposite direction, barrier dysfunction augments skin inflammation [12,13,26]. In addition to *CYP1A1* induction, AHR is an essential transcription factor for human epidermal barrier proteins including FLG and LOR [17,28,29]. Some ligands for AHR, such as coal tar and soybean tar, actively upregulate *FLG* and *LOR* expression [13,14]. In addition, barrier function is significantly disturbed in *Ahr*-null mice, indicating that AHR plays a pivotal role in skin barrier integrity [30]. The importance of FLG in the integrity of epidermal barrier function was stressed in previous studies, demonstrating that (1) loss-of-function mutation of the *FLG* gene is associated with the development of atopic dermatitis [10,11]; (2) *Flg*-deficient mice exhibit reduced epidermal barrier function with enhanced susceptibility to environmental sensitization [31]; and (3) upregulation of Flg is correlated with efficient barrier recovery of tape-stripped barrier-disrupted murine skin [32,33]. In parallel, the expression of LOR is markedly decreased in the lesional skin of atopic dermatitis and is normalized after topical treatment [13,34].

Our recent study demonstrated that OVOL1 is engaged in the AHR-mediated upregulation of *FLG* and *LOR* [18]. The present study also proved that the ligation of AHR by APE upregulated *OVOL1* expression and that knockdown of OVOL1 significantly inhibited APE/AHR-mediated *FLG* and *LOR* expression. These results indicate that the AHR/OVOL1 axis is also involved in the upregulation of *FLG* and *LOR* by APE.

## 4. Materials and Methods

### 4.1. Preparation of APE (Artemisia Princeps Extract)

Dried *Artemisia princeps* and 10 volumes of water were mixed and heated (Figure S1). This extraction process was repeated twice. The pooled water extract was spray-dried. The dried extract powder was mixed in ethanol with stirring, and the precipitated sediment was filtered and collected. The sediment was again dissolved in water and spray-dried. The dried extract powder was used as the final APE in the present study.

### 4.2. Cell Culture

Normal human epidermal keratinocytes (NHEKs) (Lifeline Cell Technology, Frederick, MD, USA) were grown in culture flasks at 37 °C in 5% CO_2_. The NHEKs were cultured in normal human epidermal keratinocyte proliferation medium, HuMedia-KG2 (KG2; Kurabo, Osaka, Japan), containing insulin, human recombinant epidermal growth factor, hydrocortisone, bovine pituitary extract, and antibacterial substances. Culture medium was replaced every one or two days. Under subconfluent (70–90% confluence) conditions, NHEKs were detached with 0.25 mg/mL trypsin containing 0.1 mg/mL ethylenediaminetetraacetic acid and subcultured.

### 4.3. Cytotoxicity Evaluation

NHEKs (2.4 × 10^4^ cells/well) were seeded on 96-well plates, allowed to attach for 48 h, and then treated with or without 0.01%, 0.03%, 0.05%, 0.07%, 0.1%, 0.3%, 0.5%, 0.7% and 1% APE for 24 h. Each well was then washed with phosphate-buffered saline (PBS) and incubated with cell proliferation reagent WST-1 (Roche, Basel, Switzerland) for 2 h at 37 °C. Thereafter, the absorbance at 450 nm was measured using an xMark™ microplate absorption spectrophotometer (BioRad, Hercules, CA, USA). The cell survival rate was calculated relative to the absorbance of the control as 100%.

### 4.4. Real-Time PCR (Polymerase Chain Reaction)

NHEKs (1.5 × 10^5^ cells/well) were seeded on 24-well cultured plates and allowed to attach for 48 h. They were then treated with or without 0.01% or 0.03% APE for 6, 18, or 24 h. Total RNA was extracted using an RNeasy^®^ Mini Kit (Qiagen, Valencia, CA, USA). Reverse transcription polymerase chain reaction (PCR) was performed using a PrimeScript RT-reagent kit (Takara Bio, Shiga, Japan). Real-time PCR was performed on an Applied Biosystems™ ViiA™ real-time PCR system (Thermo Fisher Scientific, Waltham, MA, USA) using SYBR Premix Ex Taq (Takara Bio). Initial amplification was started at 95 °C for 10 s, followed by 40 cycles of 95 °C for 5 s and 60 °C for 34 s. We used β-actin (*ACTB*) as a control housekeeping gene, because its gene expression is not affected by AHR ligation [18,35]. The sequences of primers from Sigma-Aldrich (St. Louis, MO, USA) and Takara Bio are shown in Table 1.

### 4.5. Immunofluorescence Analysis

NHEKs (7.5 × 10^4^ cells/well) were cultured on an eight-well Lab-Tek™ II Chamber Slide™ system (Nunc™; Thermo Fisher Scientific) for 24 h. They were then treated with or without 0.03% APE for 6 h. After washing with PBS, they were fixed with acetone for 10 min. After washing in PBS with 0.5 mg/mL Tween 20 (PBSt) (Sigma-Aldrich), the fixed NHEKs were treated with 100 mg/mL bovine serum albumin in PBSt for 30 min. Samples were then incubated with anti-AHR rabbit IgG (H-211; 1:50 dilution; Santa Cruz Biotechnology, Inc., Santa Cruz, CA, USA) or anti-Nrf2 polyclonal rabbit IgG antibody (H-300; 1:50 dilution; Santa Cruz) overnight at 5 °C. Next, samples were washed with PBSt and incubated with Alexa Fluor^®^ 546- or 488-conjugated anti-rabbit secondary antibody for 1 h at room temperature. Samples were then washed with PBSt and mounted with SlowFade^®^ Gold Antifade Mountant after nuclear staining with 4′,6-diamidino-2-phenylindole (DAPI). All samples were analyzed using an all-in-one fluorescence microscope (Keyence, Osaka, Japan).

### 4.6. Transfection with siRNA against AHR (Aryl Hydrocarbon Receptor)

Small interfering RNAs (siRNAs) against AHR (AHR siRNA, s1200) or OVOL1 (OVOL1 siRNA, s9939) as well as siRNA consisting of a scrambled sequence that would not lead to specific degradation of any cellular mRNA (control siRNA) were purchased from Ambion (Austin, TX, USA). NHEKs cultured in 24-well plates were incubated for 48 h in 0.5 mL of the culture medium with a mixture containing 5 nM siRNA and 3 µL of the HiPerFect Transfection reagent (Qiagen, Courtaboeuf, France). The siRNA-transfected NHEKs were further treated with 0.03% APE for 24 h. A Trypan Blue dye exclusion test was performed to assess cell viability. The inhibitory efficiency of siRNA transfections was calculated.

### 4.7. Statistical Analysis

Results are expressed as mean ± standard deviation of the mean. The unpaired Student’s *t* test was used to analyze the results using IBM SPSS Statistics 23, IBM Japan (Tokyo, Japan). In all analyses, *p* < 0.05 was taken to indicate statistical significance.

## 5. Conclusions

In conclusion, the antioxidant APE is an integral part of phytoextracts that upregulate FLG and LOR expression via AHR/OVOL1 signaling. Recently, topical agent with AHR agonistic activity has been reported to be beneficial for atopic dermatitis in clinical trial [36,37]. Targeting AHR/OVOL1/FLG or LOR signaling may be a promising strategy to overcome atopic dry skin.

## Figures and Tables

**Table 1:** Primers for PCR (Polymerase Chain Reaction).

Gene	Forward Primer	Reverse Primer
*ACTB*	5′-TTGTTACAGGAAGTCCCTTGCC-3′	5′-ATGCTATCACCTCCCCTGTGTG-3′
*CYP1A1*	5′-TAGACACTGATCTGGCTGCAG-3′	5′-GGGAAGGCTCCATCAGCATC-3′
*NQO1*	5′-GGATTGGACCGAGCTGGAA-3′	5′-AATTGCAGTGAAGATGAAGGCAAC-3′
*HO1*	5′-AAGACTGCGTTCCTGCTCAAC-3′	5′-AAAGCCCTACAGCAACTGTCG-3′
*FLG*	5′-CATGGCAGCTATGGTAGTGCAGA-3′	5′-ACCAAACGCACTTGCTTTACAGA-3′
*LOR*	5′-GGCTGCATCTAGTTCTGCTGTTTA-3′	5′-CAAATTTATTGACTGAGGCACTGG-3′
*OVOL1*	5′-ACGATGCCCATCCACTACCTG-3′	5′-TTTCTGAGGTGCTGGTCATCATTC-3′

## Supplementary Materials

Supplementary materials can be found at www.mdpi.com/1422-0067/18/9/1948/s1.
