# Supplementary material for: Antioxidant Artemisia princeps Extract Enhances the Expression of Filaggrin and Loricrin via the AHR/OVOL1 Pathway
Source: Int J Mol Sci. 2017 Sep 11;18(9):1948. doi: 10.3390/ijms18091948 (PMC5618597; doi:10.3390/ijms18091948)

Figure S1

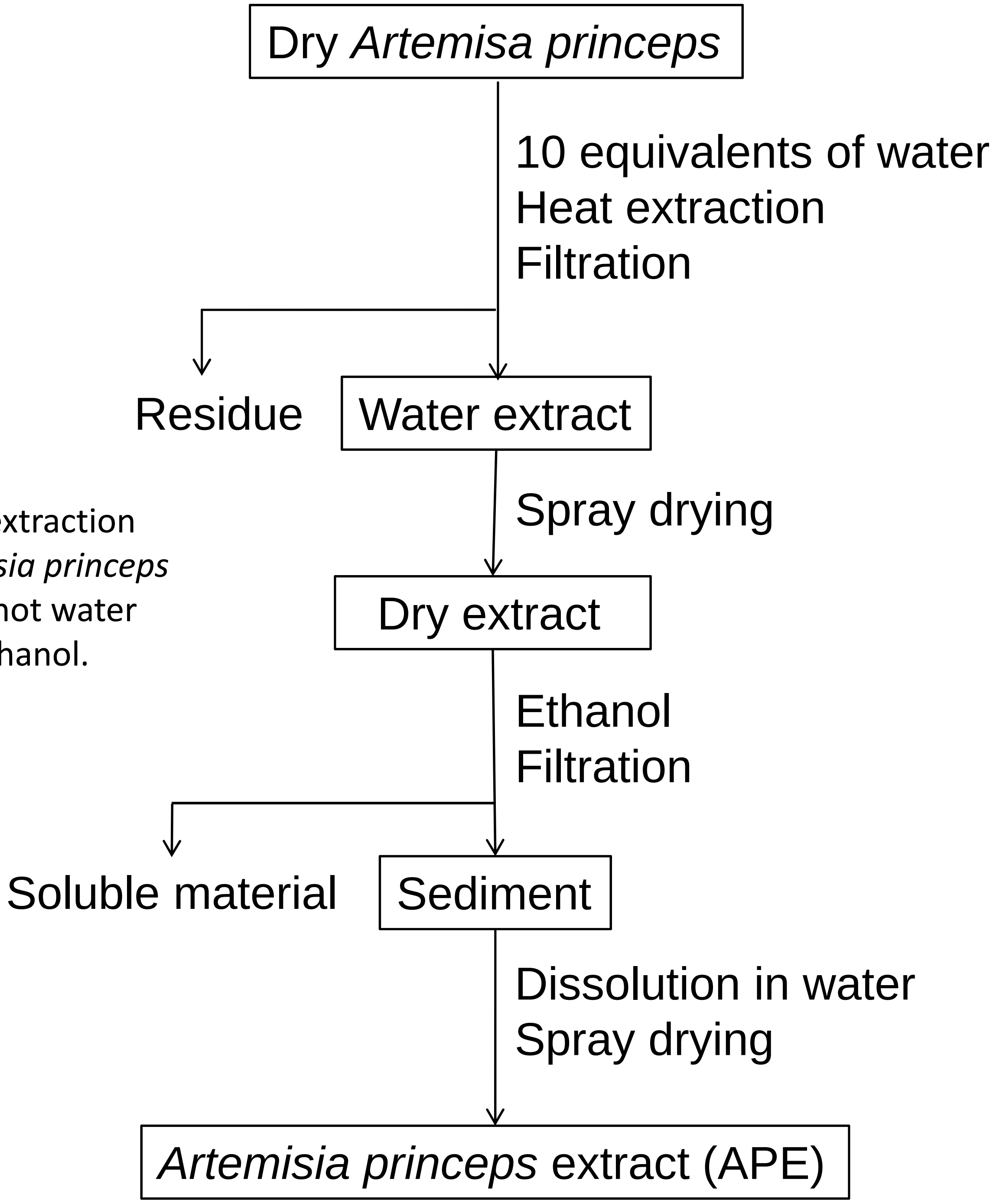

Artemisia princeps extraction process. Dry *Artemisia princeps* was extracted with hot water and purified with ethanol.

Figure S2

The viability of NHEKs was examined by cell toxicology analysis in graded concentrations of APE. NHEKs were constantly viable with APE ≤ 0.03%. Cell viability is shown as the mean ± standard deviation. **: $P < 0.01$.

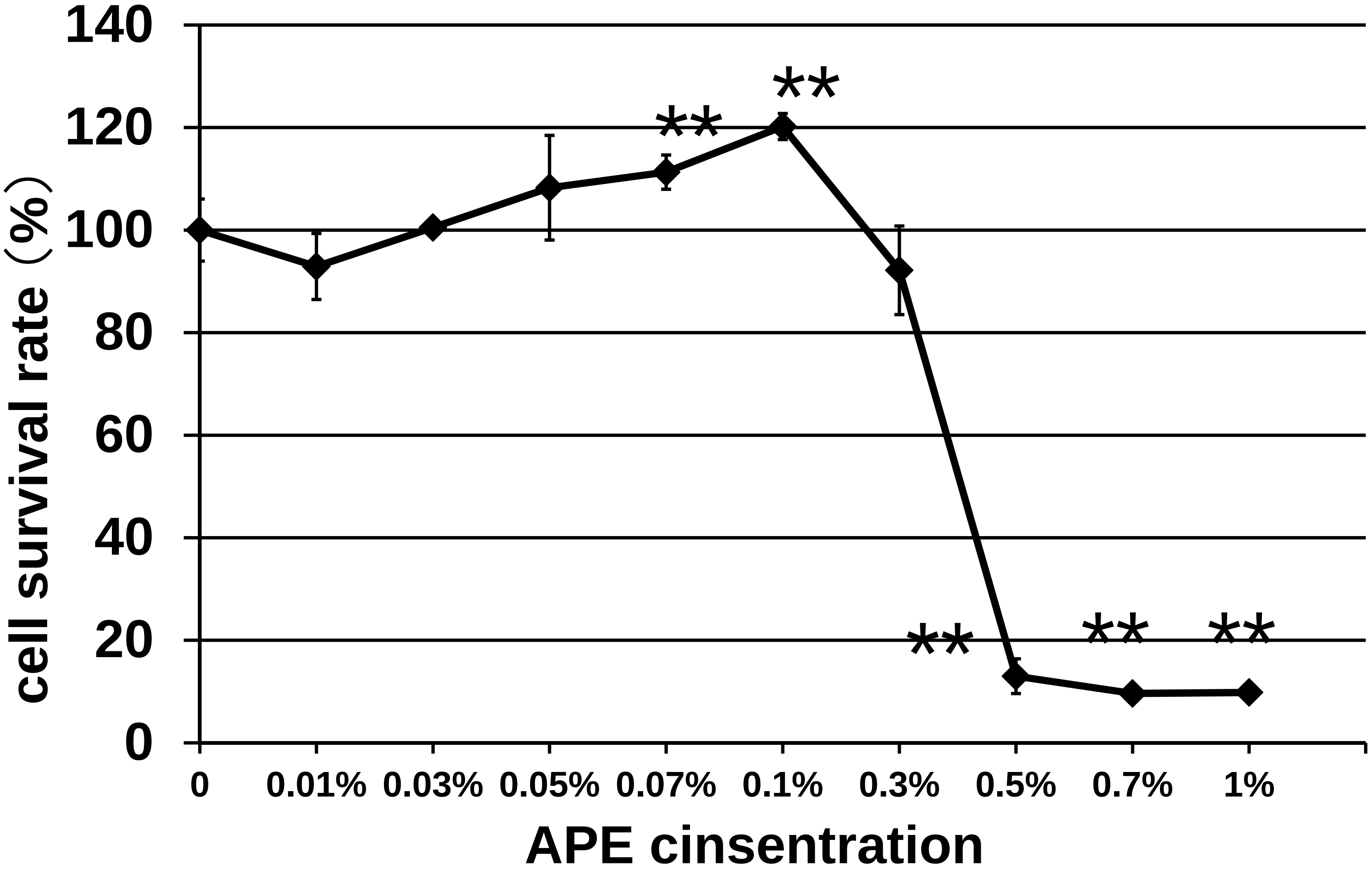

Figure S3

APE (0.03%)-induced OVOL1 upregulation is canceled in keratinocytes with AHR knockdown.
*: *P* < 0.05

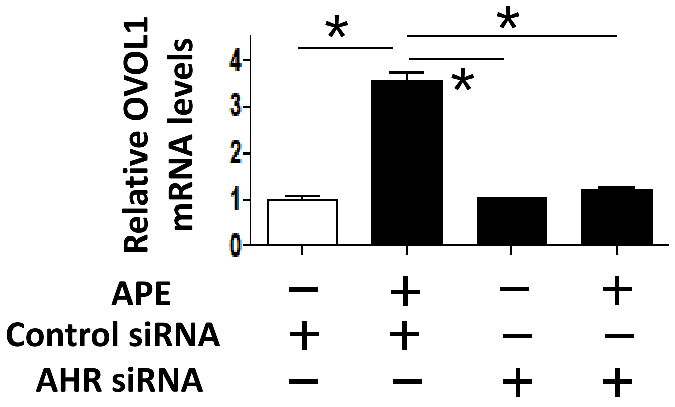

Figure S4

APE-induced NQO1 expression was only minimally downregulated in NHEKs transfected with AHR siRNA. Moreover, APE-induced HO1 expression was rather enhanced in AHR-knockdowned NHEKs. *: $P < 0.05$, **: $P < 0.01$

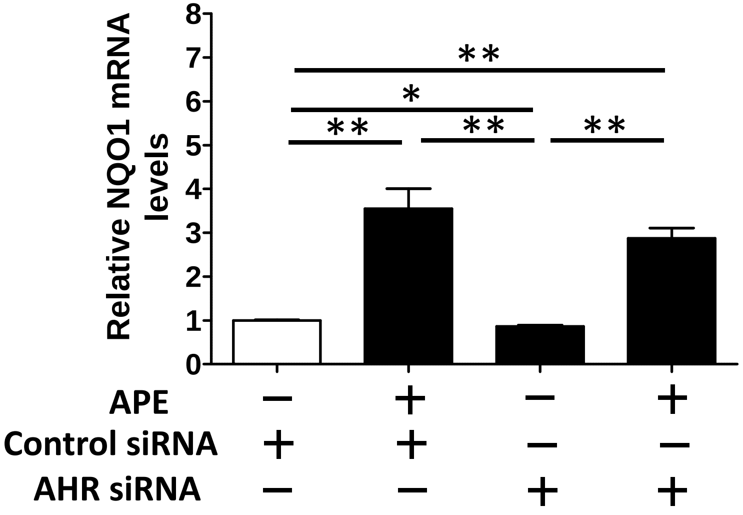

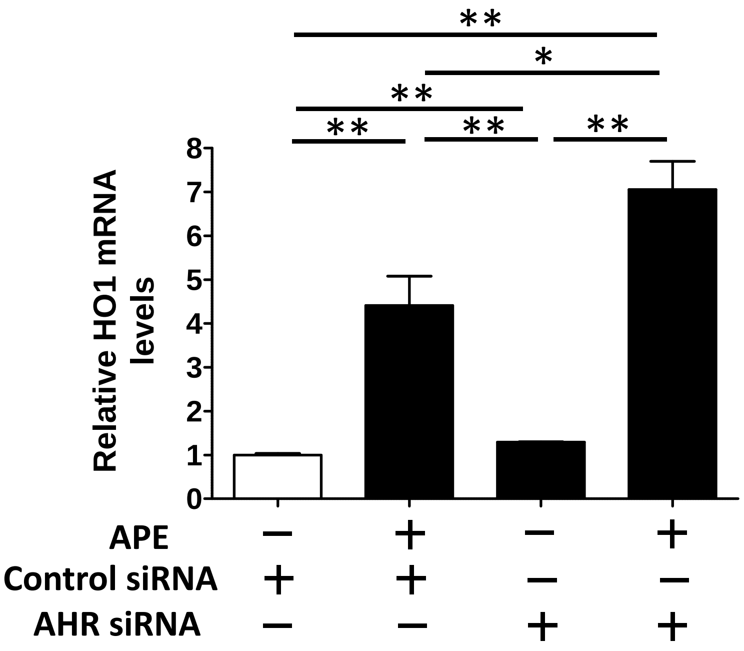

Supplement: Supplementary file 1 [file ijms-18-01948-s001.pdf]
